# P-244. Antibiotic Prescribing Patterns in Ventilator Associated Pneumonia and their Effect on Carbapenem Resistant Organism Colonisation in Asian Hospitals

**DOI:** 10.1093/ofid/ofae631.448

**Published:** 2025-01-29

**Authors:** Srishti Chhabra, Suchart Booraphun, Andrew Yunkai Li, Pornanan Domthong, Gyan Kayastha, Yie Hui Lau, Ploenchan Chetchotisakd, Direk Limmathurotsakul, Paul Tambyah, Ben S Cooper, Yin Mo

**Affiliations:** National University Health System, Singapore, Singapore; Sunpasitthiprasong Hospital, Ubon Ratchathani, Thailand, Ubon Ratchathani, Ubon Ratchathani, Thailand; Department of Intensive Care Medicine, Woodlands Health, Singapore, Singapore, Not Applicable, Singapore; Khon Kaen Hospital, Khon Kaen, Thailand, Khon Kaen, Khon Kaen, Thailand; Patan Hospital, Patan Academy of Health Sciences, Lalitpur, Nepal, Lalitpur, Bagmati, Nepal; Anaesthesiology, Intensive Care and Pain Medicine, Tan Tock Seng Hospital, Singapore, Singapore, Not Applicable, Singapore; Srinagarind Hospital, Khon Kaen University, Khon Kaen, Thailand, Khon Kaen, Khon Kaen, Thailand; Mahidol-Oxford Tropical Medicine Research Unit, Faculty of Tropical Medicine, Mahidol University, Bangkok, Thailand, Bangkok, Krung Thep, Thailand; National University Hospital, Singapore, Singapore, Not Applicable, Singapore; Centre for Tropical Medicine, Nuffield Department of Medicine, University of Oxford, Oxford, UK, Oxford, England, United Kingdom; Division of Infectious Diseases, Department of Medicine, National University Hospital, National University Health System, Singapore, Singapore, Not Applicable, Singapore

## Abstract

**Background:**

Ventilator-associated pneumonia (VAP) is associated with prolonged hospitalisation, excessive antibiotic use and high mortality. Antibiotic regimens used for VAP and their effect on development of carbapenem resistant (CR) organisms (CRO) have not been well characterised, especially in Asia.

**Table 1.**
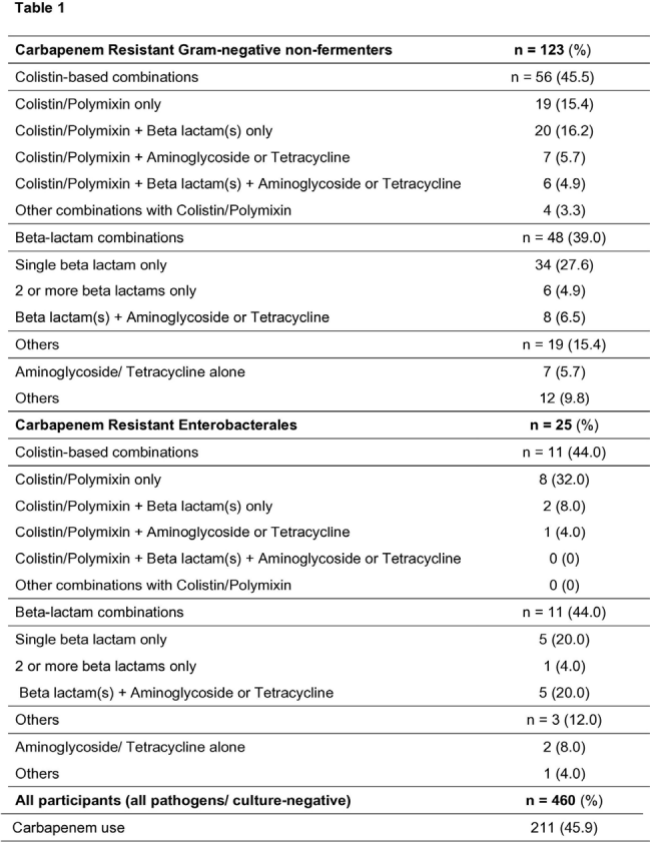
Antibiotics used for index episode of VAP caused by carbapenem resistant organisms, and overall carbapenem use

**Methods:**

The REGARD-VAP study was a randomised trial conducted in 39 intensive care units in Nepal, Singapore and Thailand. Adults aged ≥18 years with VAP (defined by the US CDC and Prevention National Healthcare Safety Network criteria), who had been mechanically ventilated for ≥48 hours, and received culture-directed antibiotics were enrolled. In culture-negative cases, antibiotics were prescribed as per local hospital antibiograms. We analysed the antibiotics prescribed in VAP and their association with CRO acquisition. We also assessed the effect of CRO colonisation on 60-day mortality and VAP recurrence.

**Table 2.**
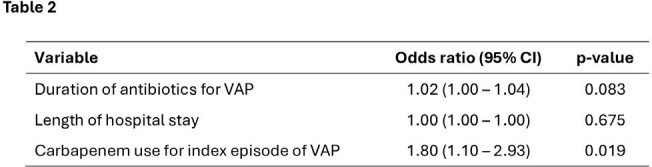
Factors associated with new carbapenem-resistant organism (CRO) isolation in microbiological samples in a multivariable logistic regression model

Carbapenem use for index episode of VAP was independently associated with higher odds ratio of new CRO colonization when adjusted for duration of antibiotics, and length of hospital stay. Duration of ICU stay was not included in this model due to high collinearity with length of hospital stay.

**Results:**

The study enrolled 460 participants and the majority (47.4%) of VAP was caused by Gram-negative non-fermenters (VAPnf). Amongst participants with CR VAPnf, 56 (45.5%) received colistin-based regimens and 48 (39.0%) received beta-lactams. Amongst those with CR Enterobacterales, 11 (44.0%) received either a colistin-based regimen or beta-lactams. 52 different antibiotic regimens were used to treat CR VAP, and only one participant received a novel beta-lactam/beta-lactamase inhibitor (Ceftazidime-avibactam). 45.9% of all antibiotic regimens included carbapenems which was independently associated with new acquisition of CRO during the study period (OR 1.80, 95% CI 1.10 – 2.93, p=0.019). In a Cox regression model, CRO colonization (n=176, 38.3%) was associated with higher 60-day mortality (HR 1.66 (95%CI 1.21 – 2.28), p=0.002) and VAP recurrence (HR 8.37 (95% CI 4.28 – 16.38), p< 0.001) when adjusted for demographic factors, disease severity, comorbidities, antibiotic duration, and VAPnf.

**Figure 1.**
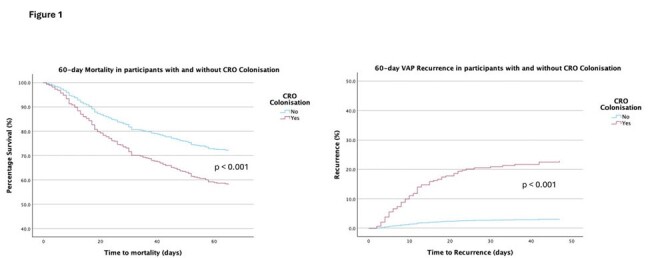
Cox-Regression Survival analysis showing 60-day mortality (left) and 60-day VAP recurrence (right) in participants with or without Carbapenem Resistant Organism (CRO) colonization.

60-day mortality and VAP recurrence was adjusted for demographic factors (patient age and gender), disease severity (qSOFA score and inotropic requirement at VAP diagnosis), comorbidities (Charlson comorbidity index and presence of congestive cardiac failure, acute coronary syndrome, chronic obstructive pulmonary disease, chronic kidney disease, cirrhosis, cancer, diabetes), duration of antibiotics prescribed for index episode of VAP, use of appropriate empiric antibiotics to which the organism was susceptible prior to switching to culture-directed antibiotics and cause of VAP (presence/ absence of Gram-negative non-fermenter). Participants with CRO colonization had increased hazards ratio of mortality and VAP recurrence.

**Conclusion:**

In lower, middle- and high-income countries in Asia, variable antibiotic regimens were used for the treatment of VAP, majority consisting of colistin and carbapenem based regimens, with the latter being associated with higher rates of new CRO isolation. There is a need for better treatment options for these difficult to treat pathogens across the income spectrum in Asia.

**Disclosures:**

**Paul Tambyah, MBBS (S'pore), Diplomate, American Board of Internal Medicine and Infectious Diseases**, Moderna: Grant/Research Support|Sanofi-Pasteur: Grant/Research Support

